# The Gluten-Free Diet: Testing Alternative Cereals Tolerated by Celiac Patients

**DOI:** 10.3390/nu5104250

**Published:** 2013-10-23

**Authors:** Isabel Comino, María de Lourdes Moreno, Ana Real, Alfonso Rodríguez-Herrera, Francisco Barro, Carolina Sousa

**Affiliations:** 1 Departamento de Microbiología y Parasitología, Facultad de Farmacia, Universidad de Sevilla, c/Profesor García González 2, 41012 Sevilla, Spain; E-Mails: icomino@us.es (I.C.); lmoreno@us.es (M.L.M.); arc@us.es (A.R.); 2 Instituto Hispalense de Pediatría, c/Guadalbullón 2, 41013 Sevilla, Spain; E-Mail: alfonsorodriguez@ihppediatria.com; 3 Instituto de Agricultura Sostenible (C.S.I.C.), Alameda del Obispo s/n, 14004 Córdoba, Spain; E-Mail: fbarro@ias.csic.es

**Keywords:** celiac disease, gluten-free diet, cereals, pseudocereals, gluten detoxification

## Abstract

A strict gluten-free diet (GFD) is the only currently available therapeutic treatment for patients with celiac disease, an autoimmune disorder of the small intestine associated with a permanent intolerance to gluten proteins. The complete elimination of gluten proteins contained in cereals from the diet is the key to celiac disease management. However, this generates numerous social and economic repercussions due to the ubiquity of gluten in foods. The research presented in this review focuses on the current status of alternative cereals and pseudocereals and their derivatives obtained by natural selection, breeding programs and transgenic or enzymatic technology, potential tolerated by celiac people. Finally, we describe several strategies for detoxification of dietary gluten. These included enzymatic cleavage of gliadin fragment by Prolyl endopeptidases (PEPs) from different organisms, degradation of toxic peptides by germinating cereal enzymes and transamidation of cereal flours. This information can be used to search for and develop cereals with the baking and nutritional qualities of toxic cereals, but which do not exacerbate this condition.

## 1. Introduction

Celiac disease is a food intolerant related syndrome that, despite being under-diagnosed, is one of the most frequent chronic gastrointestinal disorders. It develops in genetically predisposed individuals in whom unidentified environmental factors (infections, changes in microbial flora, *etc.*) can trigger intolerance to gluten contained in wheat, barley, rye and oats [1,2]. Gluten is a complex mixture of proteins called prolamins. This protein fraction has specific name: wheat prolamins are termed gliadins and glutenins, barley prolamins are hordeins, rye prolamins are secalin and those from oats are avenins. A common characteristic of these proteins is the presence of multiple proline and glutamine residues, making them resistant to gastrointestinal digestion and more exposed to deamination by tissue transglutaminase.

Several epitopes responsible for the toxicity of gluten have been identified based on their ability to stimulate proliferation of gluten-responsive T cells in celiac patient-derived small intestine biopsies. Considering only wheat, in the Immune Epitope Database (IEDB) [3] can be found 190 T-cell stimulatory epitopes related to celiac disease. Of these, 94 epitopes are located in α-gliadin genes, 74 in γ-gliadin genes, 12 in ω-gliadin genes, 8 in low molecular weight (LMW) glutenin genes, and 2 in high molecular weight (HMW) genes.

The most accepted model for explaining the immunopathogenesis of celiac disease is the two-signal model, characterized by a first innate immune response and a subsequent secondary adaptive response, which will promote a histological lesion characterized by a massive intraepithelial infiltration of lymphocytes, crypt hyperplasia and villous atrophy [2]. The ingestion of these proteins leads to the inflammation, atrophy, and hyperplasia of the small-intestinal crypts of the celiac patient. However, this disease not only affects the gut, but it is a systemic disease that may cause injury to the skin, liver, joints, brain, heart, and other organs.

Celiac disease goes in remission when the patients are put on a gluten-exclusion diet, and patients relapse when gluten is reintroduced into the diet [1,2]. Complying with a GFD is difficult and affects the patients’ quality of life, but a strict diet is critical to reduce morbidity and mortality [4].

Gluten has many special characteristics that favor its use in various food products. Because a large amount of gluten is generated during the manufacture of starch, it has a relatively low price. This may turn out to be problematic for people on a GFD, since gluten proteins may be found in unexpected sources such as meat, fish or milk products. This is the reason why alternative approaches to the GFD are actively sought [5], which include the search for and development of new cereals or gluten with no or low immunogenic content. In this article, we will review the current status of alternative cereals and their derivatives obtained by natural selection, breeding programs and transgenic or enzimatic technology, which may be potential tolerated by patients with celiac disease.

## 2. Natural Varieties of Cereal and Pseudocereals Suitable for Patients with Celiac Disease

### 2.1. Wheat and Barley

**Figure 1:**
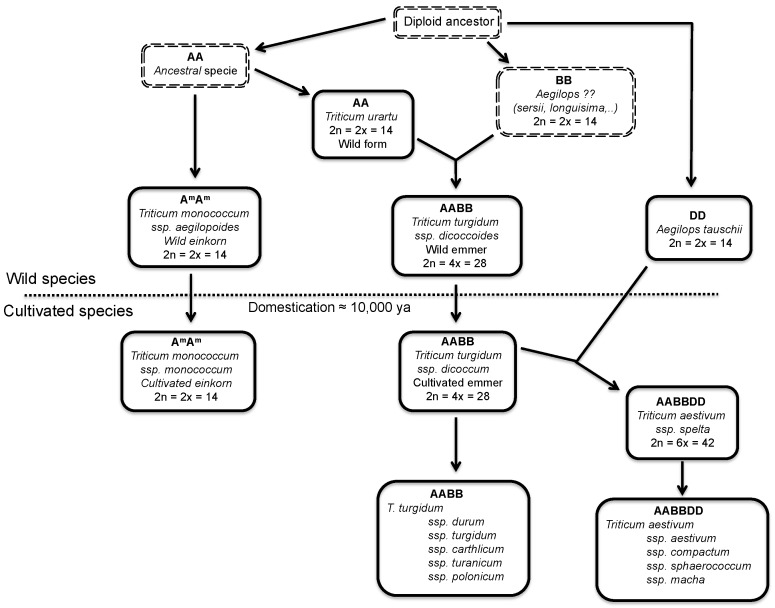
Diagram showing the evolutionary relationships among species of wheat and related species of *Aegilops*. The dotted line separates the wild and domesticated species of wheat. Ancestral or unknown species are surrounded by a double dashed rectangle. Ploidy level and the number of chromosomes are indicated. Nomenclature according to van Slageren [8].

Cultivated wheat is genetically very complex due to its origin from ancestral diploid species through a process of natural hybridization and subsequent polyploidization. The two wheat species of agricultural importance, the pasta wheat, and the bread wheat, are tetraploid (two genomes, AABB) and hexaploid (three genomes, AABBDD), respectively (Figure 1). The tetraploids originated in nature through spontaneous hybridization of two diploid species, each donor genomes A and B, between 0.5 and 2 million years ago. Bread wheat (AABBDD) originated in the fields, about 8000 years ago, through spontaneous hybridization between durum wheat (AABB) and *Aegilops tauschii*, the diploid donor of the D genome (Figure 1).

One species of wheat, *Triticum aestivum*, is predominantly used in the modern industrialized world, due to its increased protein production as well as its hardiness in colder climates. It has also been determined that the proteins that are the most immunogenic for celiac disease reside in the gliadin fraction of *T. aestivum*. However, there are almost 20 other species of wheat that are either not being cultivated by modern societies or are cultivated in select regions of the world [6]. With such a large number of wheat species available, a significant amount of research has been focused on the exploration of different species and cultivars of wheat as an alternative to a strict GFD for celiac patients [7].

Attempts have been made to quantify the toxicity of a range of bread wheat and pasta wheat varieties and of species that contain only one of the three genomes of bread wheat [9,10]. Using specific T-cell clones and monoclonal antibodies, the results demonstrated that large quantitative differences exist in the presence of toxic gluten peptides, with some cultivars completely lacking particular harmful peptides [11]. Diploid wheat species are among the suitable candidates for their low capability to activate intestinal T cell responses in celiac patients [11,12]. Compared with tetraploid and hexaploid wheat, commonly used in the making of bread and pasta, the ancient diploid *Triticum monococcum* ssp. monococcum wheat showed a marked reduction, or even a lack, of toxicity *in vitro* cellular assays [13,14]. Gianfrani *et al.* [15] compared the immunological properties of 2 lines of diploid monococcum wheat, Monlis and ID331, with those of *T. aestivum*. They found that both lines activate celiac T cell response. However, ID331 was less effective to activate the innate immune pathways. The reduced ability of some diploid wheat lines to *in vitro* activate the innate immune response in celiac mucosa could render these cultivars less active in inducing celiac disease. However, more analyses are required to explore their potential use as new dietary opportunities for celiac patients.

Some approaches were performed to remove celiac toxic proteins in barley. Double-null hybrid plants, largely devoid of both B- and C-hordeins, were produced by conventional crossing [16]. Barley is a diploid and unlike the situation in bread wheat, the genetics of hordeins are relatively straightforward. There are four protein families of hordeins: B-, C-, D- and γ-hordeins, with the B- and C-hordeins together accounting for over 90% of barley hordeins. Isolation of hordein double-null barley lines from hybrids of Risø 56 and Risø 1508 has produced a line which does not accumulate B- or C-hordein and only has 3% of wild type hordein along with a 20-fold reduction in reactivity in T-cell assays [16,17]. In addition, studies carried out demonstrated that some malting lines (*Hordeum vulgare*) were less immunogenic compared with wild lines (*Hordeum chilense*) [18]. These findings could raise the prospect of breeding barley species with low levels of harmful gluten, and the attractive goal of developing non-toxic barley cultivars with a potential use in the manufacture of beverages as consumed worldwide as are the beers. However, nothing is known about the variability in celiac toxicity of other species or varieties of toxic cereals such as rye.

### 2.2. Oats

Cultivated oats are hexaploid cereals belonging to the genus *Avena* L., which is found worldwide in almost all agricultural environments [19]. Recently, oats have been receiving increasing interest as human food, mainly because the cereal could be suitable for consumptions by celiac patients. Several varieties of oats are available. It is a rich source of protein, contains a number of important minerals, lipids, β-glucan, a mixed-linkage polysaccharide, which forms an important part of oat dietary fiber, and also contains various other phytoconstituents like avenanthramides, an indole alkaloid-gramine, flavonoids, flavonolignans, triterpenoid saponins, sterols, and tocols. Traditionally oats have been in use since long and are considered as stimulant, antispasmodic, antitumor, diuretic, and neurotonic. Oat possesses different pharmacological activities like antioxidant, anti-inflammatory, antidiabetic, anticholesterolaemic, *etc.* [20].

The presence of oats in a GFD is still a subject of controversial. Oats differ from other cereals in their prolamin content. The percentage of proline and glutamine (amino acids abundant in toxic regions) in avenin is lower than in other toxic cereals. Some clinical researchers state that patients with celiac disease tolerate oats without signs of intestinal inflammation [21]. According to the Codex Alimentarius for food for special dietary use for persons intolerant to gluten, CODEX STAN118-1979 (revised 2008, [22]), oats can be tolerated by most but not all people who are intolerant to gluten. Moreover, according to the Commission Regulation (EC) No 41/2009 [23] concerning the composition and labeling of foodstuffs suitable for people intolerant to gluten, a major concern is the contamination of oats with wheat, rye or barley that can occur during grain harvesting, transport, storage and processing. Therefore, the risk of gluten contamination in products containing oats should be taken into consideration with regard to labeling of those products. In contrast, other studies confirmed the toxicity of oats in certain types of patients with celiac disease. Arentz-Hansen *et al.* [24] described the intestinal deterioration suffered by some patients with celiac disease following the consumption of oats while on a GFD. Avenin can trigger an immunological response in these patients similar to the response produced by the gluten of wheat, rye or barley. The monitoring of 19 adult patients with celiac disease who consumed 50 g/day of oats over 12 weeks showed that one of the subjects was sensitive to oats. Therefore, it is critical to clarify either qualitatively or quantitatively the potential immunotoxicity of oats to patients with celiac disease [21,24].

Comparison of the different studies are complicated by the different study designs, the different conditions used in the testing, the number of subjects included in each study and the reporting of the purity control of the oat material used in the clinical trials. Another relevant factor in different designs is the absence of information on the oat variety used. Silano *et al.* [25] investigated the immunogenic effect of avenins from four oat cultivars using peripheral lymphocytes from patients with CD. All the varieties of oats tested (Lampton, Astra, Ava, and Nave) by these investigators were immunogenic with differences in their capacity to induce a response. However, other study confirmed that *Avena genziana* and *Avena potenza* do not display *in vitro* activities related to CD pathogenesis [26].

The utility of the G12 antibody to identify potentially toxic oat varieties for celiac patients has been reported [27]. This finding allowed classification of oat varieties into three groups based in their degree of affinity for the G12 antibody: a highly recognized group, one of moderate recognition, and one with no reactivity [27]. These results were confirmed by MALDI-TOF, SDS-PAGE and western blot by showing that the number, relative intensity of the peaks and protein profile obtained for the nine oat varieties differ from one another. The potentially immunotoxicity of the different types of oats was determined by T cell proliferation and interferon γ release. The reactivity that T-cells isolated from celiac patients exhibited with three oat varieties (one from each of the classified groups) correlated directly with the moAb G12 reactivity. The diversity observed in the reactivity to the different oat cultivars suggests variations in the avenin composition, and therefore in the amount of immunotoxic epitopes similar to the 33-mer present in these varieties. This gives a rational explanation for why only some oats trigger an immunological response.

In comparison with wheat gliadins, the avenins have been little studied, and the number of full avenin genes present at the moment in the databases is limited and from few genotypes, so that the variability of avenin genes in oats is not well represented. It has recent been known that, like wheat, oat grains have both monomeric and polymeric avenins [28]. A direct correlation between the immunogenicity of the different varieties of oats and the presence of the specific peptides with a higher/lower potential immunotoxicity has been found, that could explain why certain varieties of oats are toxic for celiac patients and other not [28]. The incorporation of some varieties of oats in food products not only may improve the nutritional quality but also may provide a treatment for various illnesses and would be welcomed by patients with celiac disease.

### 2.3. Other Cereals and Pseudocereals

It is well-known that the high nutritional value of gluten containing cereals and the viscoelastic network generated by the gluten that enables an excellent aerated structure in food products. In contrast, cereal based gluten-free products can be rich in carbohydrates and fats, and they have deficiencies in macronutrients and micronutrients. In consequence, long time adherence to GFD could induce nutrients deficiencies. Different proteins have been proposed as alternative for both playing the polymer role and increasing the nutritional value of gluten-free products. The incorporation of other ingredients/nutrients like 3-omega lipids, specific proteins, *etc.* is an alternative to improve the nutritional composition of gluten-free products.

**Figure 2:**
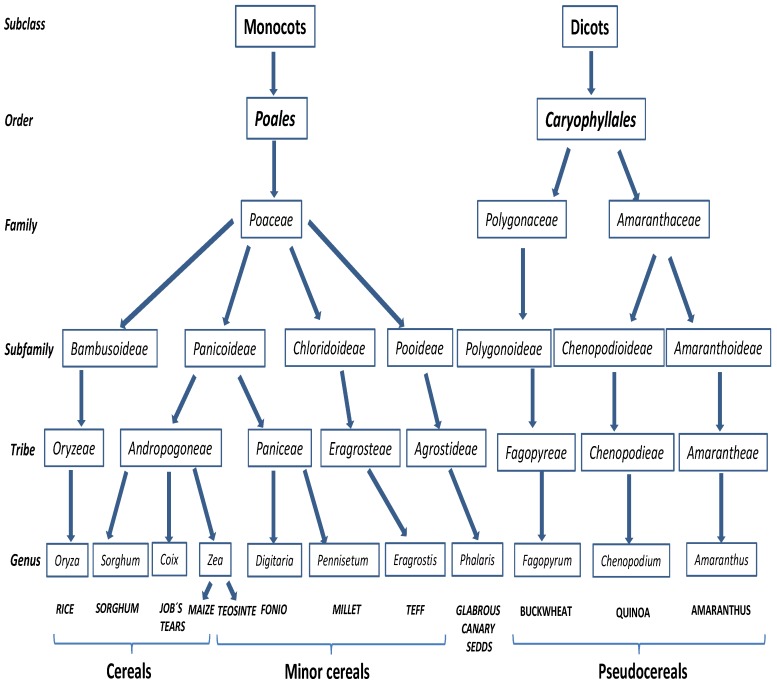
Taxonomic relation of known non-toxic cereals, minor cereals and pseudocereals in the context of celiac disease.

It is noteworthy that many grains (members of the grass family) that are closely related to wheat, rye and barley are considered toxic based on taxonomy. Furthermore, some studies focused on the protein homology in grains have supported molecular evidences [28,29]. However, member belonging to other tribes that appear to be related to corn, are considered safe (Figure 2) and can serve as substitutes and provide flours for cooking and baking for celiac and gluten-sensitive individuals. There are protein studies in support of this conclusion, although the studies are not sufficiently complete to provide more than guidance.

Non-gluten-containing sources frequently used in product formulation include cereals (rice, corn and sorghum), minor cereals (fonio, teff, millet, and job’s tears) and pseudocereals (buckwheat, quinoa and amaranth). As the environmental conditions for growing these grains are variable, availability of regular supplies is not always assured.

#### 2.3.1. Rice, Maize and Sorghum

Rice is the seed of the monocot plant of the genus *Oryza* and of the grass family Poaceae (formally Graminae), which includes twenty wild species and two cultivated ones, *Oryza sativa* (Asian rice) and *Oryza glaberrima* (African rice). Rice is one of the most important foods in the human diet and extended cereal crop. Rice is mainly consumed as white grain, but in the last decade, dozens of products containing rice as an ingredient have appeared on the food market [30]. There has been a notable increase in the use of rice flour in the formulation of gluten-free products for their hypoallergenic qualities or hypoallergenicity in spite of it is necessary to use a hydrocolloid, emulsifier, enzyme or protein to confer viscoelastic properties [30,31].

Maize (*Zea mays* subsp. *mays* L), also known as corn, is considered as a safe cereal for celiac patients. It is used as alternative to elaborate gluten-free foodstuffs. Some celiac patients considered refractory to the treatment with a GFD improved when a corn-free diet was prescribed [32]. However, some studies have showed the certain maize prolamins (zeins) contain amino acid sequences that resemble the wheat gluten immunodominant peptides and their integrity after gastrointestinal proteolysis is unknown [33]. Darewickz *et al.* [34] detected amino acid sequences with a high degree of identity to the celiac-toxic peptides in maize prolamins (zeins). This could be because the zeins, like other storage proteins, have its origin in the alpha-amylase inhibitors [35].

Sorghum (genus of numerous species of grasses) is a drought-and heat-tolerant cereal grain that grows in semiarid conditions. Whereas sorghum traditionally has been used primarily as animal feed in western countries, nearly 40% of the world sorghum production is used for human food in Africa and India. Immunological studies and *in vitro* and *in vivo* challenges of sorghum food products have supported that sorghum might provide a good basis for gluten-free foods [36]. In a recent study, Pontieri *et al.* [37] by using *in silico* approaches and biochemical/immunochemical experiments have demonstrated that sorghum can be definitively considered safe for consumption by people with celiac disease for the absence of toxic gliadin-like peptides.

#### 2.3.2. Minor Cereals

Minor cereals, so called because they are less common and are only grown in a few small regions of the world, included fonio, teff, millet, teosinte and Job’s tears [38].

Fonio (*Digitaria exilis*) is a typically cereal in Sudan or Ethiopia where it is considered to be the tastiest of all cereals [38]. Fonio can survive in poor soil conditions such as sandy and acidic soils and its composition is similar to that of other millets: limited in lysine, but rich in methionine [39].

Teff (*Eragrostis tef*) is the smallest of all grains in the world and it is classified on the basis of seed color, ranging from milky white to almost black. Teff is a cereal traditionally grown in Ethiopia and used to make *injera* or flat bread.

Millet refers to a number of different species of the *Pennisetum* genus, all of which are small-grained, annual cereal grasses. The most important type for food consumption is pearl millet that is similar in texture to rice flour [38].

Job’s tears (*Coix lacryma-jobi*), also known as Chinese Pearl Barley, is a type of millet wild tropical Asian grass related to maize. Job’s tears is naturally gluten-free, but similar to other grains it may be contaminated during processing by comingling with gluten grains such as wheat. It is used as a source of food and drinks.

#### 2.3.3. Pseudocereals

Pseudocereals are non-grasses plants which grains are used in the same way that true cereals. Pseudocereals seeds can be ground into flour and then to produce derived products like bread and pasta. Recently, the use of pseudocereals producing small grain-like seeds like amaranthus and quinoa (Amaranthaceae family), belonging to dicotyledons (Magnoliopsida class), have been considered for the preparation of gluten-free food products because the lack toxic seed proteins and have high nutritional value [40]. However, the believed lack of toxicity for most of these pseudocereals was based on their taxonomical classification rather than a direct evaluation of their inmunostimulator activity.

Several studies affirmed that amaranth and quinoa have high quality protein in terms of digestibility, efficiency ratio and nutrition balance, almost equivalent to that of milk protein casein [41,42]. Additionally, these pseudocereals are also rich in polyunsaturated fatty acids (high linolenic:linoleic acid ratio) and bioactive compounds such as γ- and β-tocopherol, polyphenols and flavonoids.

The genus *Amaranthus* L. contains more than 60 species; *A. caudatus*, *A. cruentus* and *A. hypochondriacus* are those most used for human nutrition. Amaranth proteins consist mainly of albumins and globulins, where prolamins, the toxic proteins for celiac patients, are very scarce. The essential amino acids content is high in amaranth seeds and the amino acid composition is better balanced than in most cereals. It is a good source of riboflavin, vitamin E, calcium, magnesium and irons, among minerals [40]. Studies focused on investigate from the molecular point of view the protein patterns from different amaranth cultivars to verify their suitability for the diet of subjects suffering from celiac disease, suggested that amaranth may be safely included in a GFD. However, controlled clinical studies are necessary to confirm the results and support the inclusion in the celiac’s diet [40].

Quinoa (*Chenopodium quinoa*) is an Andean grain that has been consumed for thousands of years in South America and was a staple of the Incas. There are hundreds of varieties of quinoa, ranging in color from white to red and purple to black [38]. Quinoa has a high biological value (83%) because of its high concentration of proteins (<23%), providing all of the essential amino acids [43,44,45]. Quinoa has important applications in the food and pharmaceutical industries. Due to its excellent nutritional value and a potential for production in various climates, quinoa has been classified as one of the humanity’s most promising crops [46]. Several studies to examine the suitability of quinoa for patients with celiac disease have been carried out in last years and concluded that quinoa could be a safe addition to a GFD. However, 2 cultivars had celiac-toxic epitopes that could activate the adaptive and innate immune responses in some patients with celiac disease [45]. A complete *in vivo* characterization of quinoa protein reactivity is needed to recommend their consumption by patients with celiac disease [45,47,48].

Buckwheat (*Fagopyrum* spp.) is botanically classified as a fruit and it is thought to have originated in China. It can be consumed as grains or as flour. The toasted grains are known as Kasha. Buckwheat is a highly nutritious pseudocereal known as a dietary source of protein with favorable amino acid composition and vitamins [49], starch, and dietary fiber [50], essential minerals (Steadman and others 2001), and trace elements [51]. Two species of buckwheat are cultivated for food consumption, *Fagopyrum esculentum* or common buckwheat and *Fagopyrum tartaricum* or tartary buckwheat [52]. Common buckwheat is primarily consumed in Asian countries. However, consumption in western countries including the United States is increasing due to it is the substitute for wheat flour for gluten-sensitive patients and as a health food because of its nutrient content [53,54]. It has been reported cases of buckwheat allergy in Japan, Korea and Europe [55].

#### 2.3.4. Other Cereals

An alternative grain that may potentially be considered for celiac patients is glabrous canary seed (*Phalaris canarienses* L.) that belongs to the Poaceae (Gramineae) family. In a recent study carried out by Boye *et al.* [30] confirmed that glabrous canary seeds were a good alternative gluten-free cereal and reported three techniques able to be used to support gluten-free labeling of products that contain it.

## 3. Modified Harmless Cereal Varieties

### 3.1. Gluten Detoxification by Biotechnological Methods

The use of genetic engineering to down-regulate gene expression by RNA interference (RNAi) technology [56] is now routine in many crops, including wheat, and is therefore an attractive opportunity for reducing the immunotoxic components of gluten and, hence the incidence of gluten-related allergies and intolerance in wheat. Several groups have taken advantage of the possibilities that the RNAi technology offers for the silencing of multigene families, and they have addressed the down-regulation of more than one group of gliadins and/or glutenins.

This technology was applied to down-regulated the expression of α-gliadins [57], γ-gliadins [58,59], ω-gliadins [60], all gliadins [61], and gliadins and LMW-GS [62] in bread wheat. These examples show the usefulness of RNAi to silence specific genes corresponding to gluten proteins, which are the known sources of immunogenic epitopes. However, only transgenic lines deficient in γ-gliadins [58] and in all three gliadin fractions [61] have been tested by monoclonal antibodies and T-cell assays for these transgenic lines to be used in foodstuff tolerated by many patients with celiac disease or other gluten-related pathologies.

Results reported by Gil-Humanes *et al.* [58] and Piston *et al.* [59] used two hpRNA construct to silence the γ-gliadins in two genotypes of the bread wheat cv “Bobwhite”. They reported 18 transgenic lines with reduction of the γ-gliadin fraction, from 65 to 97% depending of the transgenic line [59]. The reduction of γ-gliadins was also accompanied by an increase in other storage proteins, in particular, the ω- and α-gliadins, and the HMW-GS and LMW-GS. However, the total gliadin content did not show significant differences in the transgenic lines relative to the wild types. Later, two hpRNA constructs designed using a chimeric sequence encompassing highly conserved genes among α-, ω-, and γ-gliadins were reported [61]. They showed that the chimeric fragment was able to effectively down-regulate the expression of genes from all three gliadin groups. The gliadin composition of the transgenic lines, determined by RP-HPLC, showed a significant reduction of the gliadin content in all of the transgenic lines, ranging from 70% to 88%. Overall, the gluten proteins were decreased up to 56% while the non-gluten proteins albumins and globulins were increased in some transgenic lines [63], as consequence, the total nitrogen content of the grain was not significantly affected.

These lines hold good potential to be used in foodstuff tolerated by many patients with celiac disease or other gluten-related pathologies. The competitive ELISA system based on monoclonal antibody [64] is a good assay for quantifying the amount of gluten in foods [65]. For transgenic lines with γ-gliadins silenced [58,61], gliadin content (ppm) did not decrease significantly but increased for some lines as consequence of the compensatory effects with other gliadins, specifically α- and ω-gliadins. However, when gluten proteins from transgenic lines, deficient in all three groups of gliadins, were tested by the monoclonal antibody, there was a significant reduction of the gliadin content in all of the transgenic lines, with the average reduction of 92% and a range between 90% and 98% [61]. Total gluten proteins were extracted for T-cell assays, treated with recombinant human TG2, and tested in serial dilution for stimulation of DQ2- and DQ8-restricted T-cell clones of celiac patients [61]. The transgenic lines deficient only in γ-gliadins retained high amounts of α- and ω-gliadins, and the DQ2-γ-VII–specific T-cell clone gave strong response to the total gluten extract from those lines [61]. In contrast, a pronounced reduction in proliferative responses was seen in some transgenic lines deficient in all three gliadin fractions. There was about a 2-log reduction in the expression of the DQ2-α-II epitope in these transgenic lines. The responses of the T-cell clones specific for the other epitopes (DQ2-γ-VII, DQ8-α-I, and DQ8-γ-I) were at or below detection level for the highest concentration of gluten protein tested. They concluded that transgenic lines containing around 30% of ω-gliadin, 10% of α-gliadin, and only 1% of γ-gliadin compared with their wild-type control, were particularly inefficient to stimulate the celiac disease lesion-derived T cells [61].

One important question is how quality is affected by the silencing of gliadins, or other gluten proteins. The rheological and gluten properties, and baking quality of wheat lines with the α-gliadins down-regulated were reported [66]. They showed that the deficiency of α-gliadins did not substantially affect the baking performance of wheat flour, although breads made using flour from lines with α-gliadins silenced had lower volumes (−11%) compared to that of wild type breads. The mixing properties of 18 transgenic lines with the γ-gliadins down-regulated were determined by using the mixograph and sodium dodecyl sulfate sedimentation (SDSS) test [59]. They concluded that the reduction of γ-gliadins seems not to have a direct effect on the mixing and bread-making properties of wheat dough, but the compensatory effect on the synthesis of the other prolamins can provide stronger doughs with improved overmixing resistance. Although gliadins are not the main component affecting the bread-making quality of wheat, it is unknown the effect that the silencing of all three groups of gliadins [61], or gliadins and LMW-GS [62] will have on wheat quality. Preliminary results reported [61] based on SDSS test, showed that most of transgenic lines with ω-, γ- and α-gliadins down regulated had SDSS volumes comparable with those of wild types, and five lines had SDSS volume values significantly lower than wild types. However, SDSS volumes of these five lines were still comparable with those of the medium-quality bread wheat. The addition of non-toxic oat flour [67], or other flours from gluten-free cereals or pseudocereals, such as sorghum, buckwheat and quinoa [37,68], might compensate the lack of gliadins, enhancing the quality of the new wheat varieties.

### 3.2. Gluten Detoxification by Enzymatic Methods

Food proteins are usually degraded into small peptides and amino acids by gastric, pancreatic and brushborder enzymes. However, gluten proteins are highly resistant to complete proteolytic digestion due to their high proline and glutamine content. Since the pioneering experiments carried out by Frazer *et al.* [69], who determined that celiac-toxic proteins could be partially hydrolyzed by gastrointestinal enzymes without loss toxicity, several strategies have been considered for detoxification of dietary gluten. Some of these strategies have been based on treatment with special peptidases that hydrolyze toxic protein and peptides to nontoxic fragments.

The beginning for the enzymatic strategies was the findings that the toxicity was abolished by complete acidic hydrolysis [70]. However, researches about gluten detoxification were not developed until 21st century. The approaches included enzymatic cleavage of gliadin fragment by PEP from different organisms, degradation of toxic peptides by germinating cereal enzymes and transamidation of cereal flours [5].

#### 3.2.1. Prolyl Endopeptidases

Shan *et al.* [71] were the first in propose that PEPs could catalyze breakdown of gluten peptides and thereby diminish its toxic effects. This hypothesis was based on that the abundance and location of proline residues is a crucial factor for the gastrointestinal resistance, and the unique ability of these enzymes to hydrolyze the peptide bond on the carboxyl side of a proline residue. Since then, further studies have shown that the fermentation of wheat, rye and barley flours with selected peptidases cause a significant decrease of gluten toxicity. The PEPs are widely distributed in bacteria, fungi, animals and plants, but it is known that lactic acid bacteria (lactobacilli) have a very complex peptidase system [72]. Lactobacilli species isolated from sourdoughs have been screened with respect to gluten degradation, finding that a pool of peptidases is needed to degraded α-gliadin fragments [73]. Studies based on use lactobacillus as microbial inoculum during fermentation of flour mixture has shown a potential ability to hydrolyze wheat prolamins by *in vitro* and *in vivo* assays [74]. Despite that, the gluten concentration remains high, therefore studies based on more complex formulas were developed. Recently, the combination of lactobacilli and fungal peptidases has been selected to eliminate the toxicity of wheat flour during long-time fermentation. Thus, food processing by selected proteases opens new perspectives toward an efficient approach to eliminate gluten toxicity.

#### 3.2.2. Germinating Cereals

The role of the proline- and glutamine-rich storage proteins of cereals is to supply the embryo with nitrogen and amino acids during the first period of seedling development. Therefore, it is likely that endogenous cereal proteases synthesized during germination would be capable of extensively hydrolyzing these proteins [75]. Given evidences about this capacity, the use of proteases from germinating wheat seeds was proposed to create safe cereal products for celiac patients [76,77]. The analysis of protein content by RP-HPLC during kernels germination of wheat, rye and barley demonstrated a remarkable degradation of prolamins. In further experiments, protease extracts from these germinated cereals cleave peptides rapidly into non-toxic fragments with less than nine amino acids [75]. Comparative studies of proteases efficacy from different cereals by *in vitro* models have revealed that barley enzymes were superior in diminish the toxicity of gliadin and secalin, but there were only minor differences between the three enzyme mixtures (oats, wheat and barley) [78]. On the other hand, it should be pointed out that germinating cereal proteases have distinct advantages in comparison to bacterial and fungal peptidases. These enzymes derive from a naturally safe food source being excellent alternatives to recombinant proteases, which might not be accepted by many celiac patients. Indeed, the production of germinated cereals, just like the extraction of highly active proteases, is simple and well-established technological process [75]. Altogether, these enzymes from germinating cereals might be utilized in food processing to develop high quality food safe for celiac patients.

#### 3.2.3. Transamidation

The enzyme transglutaminase (TG) catalyzes two classes of reactions, transamidation and deamidation. The first one transforms the primary γ-carboxamide group to a secondary one, and in the second reaction, the glutaminyl residue is converted to a glutamyl residue [79]. Tissue transglutaminase 2 (tTG) has been describe as one of the key factor in the immunopathogenesis of celiac disease because of gluten peptides increase their immunogenicity due to the deamidation [80]. Considering that only the transamidation might be use for gluten detoxification, several studies have been focused to know the ratio between these two reactions [81,82]. It seems that the ratio can vary considerably depending on different factors like the presence of primary amines, the peptide sequence as well as enzyme concentration. In general terms, it has been found highest rate of deamidation in tTG, however, the few studies carry out with TGs from other origins have shown a considerably lower deamidation *versus* transamidation activity in microbial TG (mTG) [83,84]. Based on that, Gianfrani *et al.* [85] treated wheat flour with mTG and lysine methyl ester to abolish gluten activity, detecting the decrease of the activity mediated by T-cell. Thus, they suggested a food-grade enzyme and an appropriate amine donor to block the T cell-mediated gliadin activity [85]. Recently, Mazzarella *et al.* [86] has shown in a randomized single blinded trial that transamidated gluten reduced the number of clinical relapses in challenged patients with no changes of baseline values for serological/mucosal celiac markers and an unaltered kidney function. Other application has been use the mTG from *S. mobaraensis* to detoxify cereal-based beverages since the beer treatment with mTG will lead to crosslinking of residual gluten peptide. If that aggregate exceeds a certain MW, they lose their solubility and can be removed from the beer resulting in beverages with gluten content below 20 mg/kg [87]. Therefore, although mTG is not able to degrade gluten, it could be used to immunodetoxify gluten.

## 4. Conclusions

Currently the only treatment for celiac disease is a lifelong GFD. However, adherence to the GFD is not easy, due to the ubiquitous nature of gluten, cross-contamination of foods and social constraints. While many patients are content with their GFD, others would welcome alternative treatments and/or food products that would allow more flexibility.

Here, we review the status of potential alternative cereals and pseudocereals and their derivatives under consideration for celiac disease. Diploid wheat species appear to be among the suitable candidates for their low capability to activate intestinal T cell responses in celiac patients. Compared with tetraploid and hexaploid wheats commonly used in the making of bread and pasta, the ancient diploid *Triticum monococcum* ssp. monococcum wheat showed a marked reduction of toxicity *in vitro* assays. Moreover, the use of genetic engineering to down-regulate gene expression by RNAi technology represents an attractive opportunity for reducing the immunotoxic components of wheat. Simultaneous silencing of the full complement of gliadins results effective for the reduction of T-cell epitopes in celiac disease.

Several studies have been demonstrated that oat immunogenicity for patients with celiac disease varies according to the cultivars. The incorporation of some varieties of oats in food products not only may improve the nutritional quality, but may provide a treatment for various illnesses and would be welcomed by patients with celiac disease.

Non-gluten-containing sources frequently used in product formulation include cereals (rice, corn and sorghum), minor cereals (fonio, teff, millet, and job’s tears) and pseudocereals (buckwheat, quinoa and amaranth). However, new studies seem to show that certain cereals and pseudocereals such as corn and quinoa, traditionally considered safe for celiac patients, could activate the immune response in some celiac patients.

More recently, studies have been showed several strategies for detoxification of dietary gluten based on treatment with special peptidases that hydrolyze toxic protein and peptides to nontoxic fragments. These included enzymatic cleavage of gliadin fragment by PEPs from different organisms, degradation of toxic peptides by germinating cereal enzymes and transamidation of cereal flours. Food processing by selected proteases opens new perspectives toward an efficient approach to eliminate gluten toxicity, which could allow the development of foods with reduced or absent levels of gluten.

## References

[1] Sollid L.M. (2002). Coeliac disease: Dissecting a complex inflammatory disorder. Nat. Rev. Immunol..

[2] Bernardo D., Peña A.S. (2012). Developing strategies to improve the quality of life of patients with gluten intolerance in patients with and without coeliac disease. Eur. J. Intern. Med..

[3] Immune Epitope Database and Analysis Resourse. http://www.iedb.org/.

[4] Corrao G., Corazza G.R., Bagnardi V., Brusco G., Ciacci C., Cottone M., Sategna Guidetti C., Usai P., Cesari P., Pelli M.A. (2001). Mortality in patients with coeliac disease and their relatives: A cohort study. Lancet.

[5] Rashtak S., Murray J.A. (2012). Review article: Coeliac disease, new approaches to therapy. Aliment. Pharmacol. Ther..

[6] Feldman M., Levy A.A. (2009). Genome evolution in allopolyploid wheat—A revolutionary reprogramming followed by gradual changes. J. Genet. Genomics.

[7] Marietta E.V., Murray J.A. (2012). Testing the safety of alternative wheat species and cultivars for consumption by celiac patients. Am. J. Clin. Nutr..

[8] Van Slageren M.W. (1994). Wild wheats: A Monograph of Aegilops L. and Amblyopyrum (Jaub. & Spach) Eig (Poaceae).

[9] Auricchio S., de Ritis G., de Vincenzi M., Occorsio P., Silano V. (1982). Effects of gliadin-derived peptides from bread and durum wheats on small intestine cultures from rat fetus and coeliac children. Pediatr. Res..

[10] Van Herpen T.W., Goryunova S.V., van der Schoot J., Mitreva M., Salentijn E., Vorst O., Schenk M.F., van Veelen P.A., Koning F., van Soest L.J. (2006). Alpha-Gliadin genes from the A, B, and D genomes of wheat contain different sets of celiac disease epitopes. BMC Genomics.

[11] Spaenij-Dekking L., Kooy-Winkelaar Y., van Veelen P., Drijfhout J.W., Jonker H., van Soest L., Smulders M.J., Bosch D., Gilissen L.J., Koning F. (2005). Natural variation in toxicity of wheat: Potential for selection of nontoxic varieties for celiac disease patients. Gastroenterology.

[12] Molberg O., Uhlen A.K., Jensen T., Flaete N.S., Fleckenstein B., Arentz-Hansen H., Raki M., Lundin K.E., Sollid L.M. (2005). Mapping of gluten T-cell epitopes in the bread wheat ancestors: Implications for celiac disease. Gastroenterology.

[13] Pizzuti D., Buda A., D’Odorico A., D’Incà R., Chiarelli S., Curioni A., Martines D. (2006). Lack of intestinal mucosal toxicity of *Triticum monococcum* in coeliac disease patients. Scand. J. Gastroenterol..

[14] Vincentini O., Maialetti F., Gazza L., Silano M., Dessi M., de Vincenzi M., Pogna N.E. (2007). Environmental factors of coeliac disease: Cytotoxicity of hulled wheat species *Triticum monococcum*, *T. turgidum* ssp. *dicoccum* and *T. aestivum* ssp. *spelta*. J. Gastroenterol. Hepatol..

[15] Gianfrani C., Maglio M., Rotondi Aufiero V., Camarca A., Vocca I., Iaquinto G., Giardullo N., Pogna N., Troncone R., Auricchio S. (2012). Immunogenicity of monococcum wheat in celiac patients. Am. J. Clin. Nutr..

[16] Tanner G.J., Howitt C.A., Forrester R.I., Campbell P.M., Tye-Din J.A., Anderson R.P. (2010). Dissecting the T-cell response to hordeins in coeliac disease can develop barley with reduced immunotoxicity. Aliment. Pharmacol. Ther..

[17] Tanner G.J., Blundell M.J., Colgrave M.L., Howitt C.A. (2013). Quantification of hordeins by ELISA: The correct standard makes a magnitude of difference. PLoS One.

[18] Comino I., Real A., Gil-Humanes J., Pistón F., de Lorenzo L., Moreno M.L., López-Casado M.Á., Lorite P., Cebolla A., Torres M.I. (2012). Significant differences in coeliac immunotoxicity of barley varieties. Mol. Nutr. Food. Res..

[19] Suttie J.M., Reynolds S.G. Fodder oats: A world overview, 2004. Food and Agriculture Organization of the United Nations (FAO). http://www.fao.org/docrep/008/y5765e/y5765e00.htm.

[20] Eppendorfer W.H. (2006). Nutritive value of oat and rye grain protein as influenced by nitrogen and amino acid composition. J. Sci. Food Agric..

[21] Pulido O., Gillespie Z., Zarkadas M., Dubois S., Vavasour E., Rashid M., Switzer C., Godefroy S.B. (2009). Introduction of oats in the diet of individuals with coeliac disease: A systematic review. Adv. Food Nutr. Res..

[22] Codex Alimentarius International Food Standars. http://www.codexalimentarius.net/web/more_info.jsp?id_sta=291.

[23] (2009). COMMISSION REGULATION (EC) No 41/2009 of 20 January 2009 Concerning the Composition and Labelling of Foodstuffs Suitable for People Intolerant to Gluten. http://eur-lex.europa.eu/LexUriServ/LexUriServ.do?uri=OJ:L:2009:016:0003:0005:EN:PDF.

[24] Arentz-Hansen H., Fleckenstein B., Molberg Ø., Scott H., Koning F., Jung G., Roepstorff P., Lundin K.E., Sollid L.M. (2004). The molecular basis for oat intolerance in patients with coeliac disease. PLoS Med..

[25] Silano M., di Benedetto R., Maialetti F., de Vincenzi A., Calcaterra R., Cornell H.J., de Vincenzi M. (2007). Avenins from different cultivars of oats elicit response by coeliac peripheral lymphocytes. Scand. J. Gastroenterol..

[26] Maglio M., Mazzarella G., Barone M.V., Gianfrani C., Pogna N., Gazza L., Stefanile R., Camarca A., Colicchio B., Nanayakkara M. (2011). Immunogenicity of two oat varieties, in relation to their safety for celiac patients. Scand. J. Gastroenterol..

[27] Comino I., Real A., de Lorenzo L., Cornell H., López-Casado M.Á., Barro F., Lorite P., Torres M.I., Cebolla A., Sousa C. (2011). Diversity in oat potential immunogenicity: Basis for the selection of oat varieties with no toxicity in celiac disease. Gut.

[28] Real A., Comino I., de Lorenzo L., Merchán F., Gil-Humanes J., Giménez M.J., López-Casado M.Á., Torres M.I., Cebolla Á., Sousa C. (2012). Molecular and immunological characterization of gluten proteins isolated from oat cultivars that differ in toxicity for celiac disease. PLoS One.

[29] Tye-Din J.A., Stewart J.A., Dromey J.A., Beissbarth T., van Heel D.A., Tatham A., Henderson K., Mannering S.I., Gianfrani C., Jewell D.P. (2010). Comprehensive, quantitative mapping of T cell epitopes in gluten in celiac disease. Sci. Transl. Med..

[30] Rosell C.M., Marco C., Arendt E.K., dal Bello F. (2008). Rice. Gluten Free Cereal Products and Beverages.

[31] Boye J.I., Achouri A., Raymond N., Cleroux C., Weber D., Koerner T.B., Hucl P., Patterson C.A. (2013). Analysis of Glabrous canary seeds by ELISA, Mass spectrometry, and western blotting for the absence of cross-reactivity with major plant food allergens. J. Agric. Food Chem..

[32] Accomando S., Albino C., Montaperto D., Amato G.M., Corsello G. (2006). Multiple food intolerance or refractory celiac sprue?. Dig. Liver Dis..

[33] Cabrera-Chávez F., Iamett S., Miriani M., Calderón de la Barca A.M., Mamone G., Bonomi F. (2012). Maize prolamins resistant to peptic-tryptic digestion maintain immune-recognition by IgA from some celiac disease patients. Plant Foods. Hum. Nutr..

[34] Darewicz M., Dziuba J., Minkiewicz P. (2007). Computational characterization and identification of peptides for in silico detection of potentially celiac-toxic proteins. Food Sci. Technol. Int..

[35] Shewry P.R., Napier J.A., Tatham A.S. (1995). Seed storage proteins: Structures and biosynthesis. Plant Cell.

[36] Ciacci C., Maiuri L., Caporaso N., Bucci C., del Giudice L., Rita Massardo D., Pontieri P., di Fonzo N., Bean S.R., Ioerger B. (2007). Celiac disease: *in vitro* and *in vivo* safety and palatability of wheat-free sorghum food products. Clin. Nutr..

[37] Pontieri P., Mamone G., de Caro S., Tuinstra M.R., Roemer E., Okot J., de Vita P., Ficco D.B., Alifano P., Pignone D. (2013). Sorghum, a healthy and gluten-free food for celiac patients as demonstrated by genome, biochemical, and immunochemical analyses. J. Agric. Food. Chem..

[38] Saturni L., Ferretti G., Bacchetti T. (2010). The gluten-free diet: Safety and nutritional quality. Nutrients.

[39] Arendt E.K., Dal Bello F. (2011). Gluten-Free Cereal, Products and Beverages (Food Science and Technology).

[40] Ballabio C., Uberti F., di Lorenzo C., Brandolini A., Penas E., Restani P. (2011). Biochemical and immunochemical characterization of different varieties of amaranth (*Amaranthus L.* ssp.) as a safe ingredient for gluten-free products. J. Agric. Food. Chem..

[41] Ranhotra G.S., Gelroth J.A., Glaser B.K., Lorenz K.J., Johnson D.L. (1993). Composition and protein nutritional quality of quinoa. Cereal. Chem..

[42] Repo-Carrasco R., Esponiza C., Jacobsen S.-E. (2003). Nutritional value and use of the Andean crops: Quinoa (*Chenopodium quinoa*) and kañiwa (*Chenopodium pallidicaule*). Food Rev. Int..

[43] Abugoch J.L.E., Taylor S.L. (2009). Quinoa (*Chenopodium quinoa* Wild.): Composition, Chemistry, Nutritional, and Functional Properties. Advances in Food and Nutrition Research.

[44] Gonzalez J.A., Konishi Y., Bruno M., Valoy M., Prado F.E. (2012). Interrelationships among seed yield, total protein and amino acid composition of ten quinoa (*Chenopodium quinoa*) cultivars from two different agroecological regions. J. Sci. Food Agric..

[45] Zevallos V.F., Ellis H.J., Suligoj T., Herencia L.I., Ciclitira P.J. (2012). Variable activation of immune response by quinoa (*Chenopodium quinoa* Wild.) prolamins in celiac disease. Am. J. Clin. Nutr..

[46] Mäkinen O.E., Zannini E., Arendt E.K. (2013). Germination of oat and quinoa and evaluation of the malts as gluten free baking ingredients. Plant Foods Hum. Nutr..

[47] De Vincenzi M., Silano M., Luchetti R., Carratu B., Boniglia C., Pogna N.E. (1999). Agglutinating activity of alcohol-soluble proteins from quinoa seed flour in celiac disease. Plant Foods Hum. Nutr..

[48] Berti C., Ballabio C., Restani P., Porrini M., Bonomi F., Iametti S. (2004). Immunochemical and molecular properties of proteins in *Chenopodium quinoa*. Cereal Chem..

[49] Bonafaccia G., Marocchini M., Kreft I. (2003). Composition and technological properties of the flour and bran from common and tartary buckwheat. Food Chem..

[50] Skrabanja V., Kreft I., Golob T., Modic M., Ikeda S., Ikeda K., Kreft S., Bonafaccia G., Knapp M., Kosmelj K. (2004). Nutrient content in buckwheat milling fractions. Cereal Chem..

[51] Bonafaccia G., Gambelli L., Fabjan N., Kreft I. (2003). Trace elements in flour and bran from common and Tartary buckwheat. Food Chem..

[52] Ikeda K. (2002). Buckwheat: Composition, chemistry and processing. Adv. Food Nutr. Res..

[53] Stember R.H. (2006). Buckwheat allergy. Allergy Asthma Proc..

[54] Wieslander G. (1996). Review on buckwheat allergy. Allergy.

[55] Panda R., Taylor S.L., Goodman R.E. (2010). Development of a Sandwich Enzyme-Linked Immunosorbent Assay (ELISA) for detection of buckwheat residues in food. J. Food Sci..

[56] Watanabe Y., Kodama H., Komamine A. (2011). Overview of Plant RNAi. Methods in Molecular Biology.

[57] Becker D., Wieser H., Koehler P., Folck A., Mühling K.H., Zörb C. (2012). Protein composition and techno-functional properties of transgenic wheat with reduced α-gliadin content obtained by RNA interference. J. Appl. Bot. Food Qual..

[58] Gil-Humanes J., Pistón F., Hernando A., Álvarez J.B., Shewry P.R., Barro F.  (2008). Silencing of γ-gliadins by RNA interference (RNAi) in bread wheat. J. Cereal Sci..

[59] Pistón F., Gil-Humanes J., Rodríguez-Quijano M., Barro F. (2011). Down-Regulating γ-gliadins in bread wheat leads to non-specific increases in other gluten proteins and has no major effect on dough gluten strength. PLoS One.

[60] Altenbach S.B., Allen P.V. (2011). Transformation of the US bread wheat “Butte 86” and silencing of omega-5 gliadin genes. GM Crops.

[61] Gil-Humanes J., Pistón F., Tollefsen S., Sollid L.M., Barro F. (2010). Effective shutdown in the expression of celiac disease-related wheat gliadin T-cell epitopes by RNA interference. Proc. Natl. Acad. Sci. USA.

[62] Wen S., Wen N., Pang J., Langen G., Brew-Appiah R.A.T., Mejias J.H., Osorio C., Yang M., Gemini R., Moehs C.P. (2012). Structural genes of wheat and barley 5-methylcytosine DNA glycosylases and their potential applications for human health. Proc. Natl. Acad. Sci. USA.

[63] Gil-Humanes J., Pistón F., Shewry P.R., Tosi P., Barro F. (2011). Suppression of gliadins results in altered protein body morphology in wheat. J. Exp. Bot..

[64] Valdés I., García E., Llorente M., Méndez E. (2003). Innovative approach to low-level gluten determination in foods using a novel sandwich enzyme-linked immunosorbent assay protocol. Eur. J. Gastroenterol. Hepatol..

[65] Codex Alimentarius International Food Standars. http://www.codexalimentarius.org.

[66] Wieser H., Koehler P., Folck A., Becker D., Lookhart L.G., Ng W.P.K. (2006). Characterization of Wheat with Strongly Reduced α-Gliadin Content. Gluten Proteins.

[67] Van den Broeck H.C., Gilissen L.J.W.J., Smulders M.J.M., van der Meer I.M., Hamer R.J. (2011). Dough quality of bread wheat lacking alpha-gliadins with celiac disease epitopes and addition of celiac-safe avenins to improve dough quality. J. Cereal Sci..

[68] Alvarez-Jubete L., Auty M., Arendt E.K., Gallagher E. (2010). Baking properties and microstructure of pseudocereal flours in gluten-free bread formulations. Eur. Food Res. Technol..

[69] Frazer A.C., Fletcher R.F., Ross C.A.C., Shaw B., Sammons H.G., Schneider R. (1959). Gluten-Induce enteropathy the effect of partially digested gluten. Lancet.

[70] Van de Kamer J.H., Weijers H.A. (1955). Celiac disease. V. Some experiments on the cause of the harmful effect of wheat gliadin. Acta Pediatr. Scand..

[71] Shan L., Molberg Ø., Parrot I., Hausch F., Gray G.M., Sollid L.M., Khosla C. (2002). Structural basis for gluten intolerance in celiac sprue. Science.

[72] Di Cagno R., de Angelis M., Lavermicocca P., de Vincenzi M., Giovanini C., Faccia M., Gobbetti M. (2002). Proteolysis by sourdough lactic acid bacteria: Effects on wheat flour protein fractions and gliadin peptides involved in human cereal intolerance. Appl. Environ. Microbiol..

[73] Gerez C.L., Font de Valdez G., Rollan G.C. (2008). Functionality of lactic acid bacteria peptidase activities in the hydrolysis of gliadin-like fragments. Appl. Microbiol..

[74] Di Cagno R., de Angelis M., Auricchio S., Greco L., Clarke C., de Vincenzi M., Giovanini C., D’Archivio M., Landolfo F., Parrilli G. (2004). Sourdough bread made from wheat and nontoxic flours and started with selected lactobacilli is tolerated in celiac sprue patients. Appl. Environ. Microbiol..

[75] Hartmann G., Koehler P., Wieser H. (2006). Rapid degradation of gliadin peptides toxic for celiac disease patients by proteases from germinating cereals. J. Cereal Sci..

[76] Kiyosaki T., Matsumoto I., Asakura T., Funaki J., Kuroda M., Misaka T., Arai S., Abe K. (2007). Gliadain, a gibberellin-inducible cysteine proteinase occurring in germinating seeds of wheat, *Triticum aestivum* L., specifically digests gliadin and is regulated by intrinsic cystatins. FEBS J..

[77] Michalcova E., Potoka E., Chmelova D., Ondrejovic M. (2012). Study of wheat protein degradation during germination. J. Microbiol. Biotechnol..

[78] Stenman S.M., Lindfors K., Venäläinen J.I., Hautala A., Mänistö P.T., Garcia-Horsman J.A., Kaukorvita-Norja A., Auriola S., Mauriala T., Mäki M. (2010). Degradation of celiac disease-inducing rye secalin by germinating cereal enzymes: Diminishing toxic effects in intestinal epithelial cells. Clin. Exp. Immunol..

[79] Wieser H., Koehler P. (2012). Detoxification of gluten by means of enzymatic treatment. J. AOAC Int..

[80] Van de Wal Y., Kooy Y., van Veelen P.A., Pena S., Mearin L., Papadopoulus G., Koning F. (1998). Selective deamidation by tissue transglutaminase strongly enhances gliadin-specific T cell reactivity. J. Immunol..

[81] Skovbjerg H., Koch C., Anthonsen D., Sjoestroem H. (2004). Deamidation and cross-linking of gliadin peptides by transglutaminases and the relation to celiac disease. Biochim. Biophys. Acta.

[82] Stamnaes J., Fleckenstein B., Sollid L.M. (2008). The propensity for deamidation and transamidation of peptides by transglutaminase 2 is dependent on substrate affinity and reaction conditions. Biochim. Biophys. Acta.

[83] Ohtsuka T., Umezawa Y., Nio N., Kubota K. (2001). Comparison of deamidation activity of transglutaminases. J. Cereal Sci..

[84] Nonaka M., Sawa A., Matsuura Y., Motoki M., Nio N. (1996). Deamidation of several food proteins using free and immobilized Ca^2+^-independent microbial transglutaminase. Biosci. Biotechnol. Biochem..

[85] Gianfrani C., Siciliano R.A., Facchiano A.M., Camarca A., Mazzeo M.F., Costantini S., Salvati V.M., Maurano F., Mazzarella G., Iaquinto G. (2007). Transamidation of wheat flour inhibits the response to gliadin of intestinal T cells in celiac disease. Gastroenterology.

[86] Mazzarella G., Salvati V.M., Laquinto G., Stefanile R., Capobianco F., Luongo D., Bergamo P., Maurano F., Giardullo N., Malamisura B. (2012). Reintroduction of gluten following flour transamidation in adult celiac patients: A randomized, controlled clinical study. Clin. Dev. Immunol..

[87] Pasternack R., Marx S., Jordan D. (2006). Prolamin-Reduced Beverages and Methods for the Preparation Thereof. Int. Pat..

